# Navigating the vestibular maze: text-mining analysis of publication trends over five decades

**DOI:** 10.3389/fneur.2024.1292640

**Published:** 2024-03-15

**Authors:** Amit Wolfovitz, Nir A. Gecel, Yoav Gimmon, Shaked Shivatzki, Vera Sorin, Yiftach Barash, Eyal Klang, Idit Tessler

**Affiliations:** ^1^Department of Otolaryngology and Head and Neck Surgery, Sheba Medical Center, Tel-Hashomer, Israel; ^2^Faculty of Medicine, Tel Aviv University, Tel Aviv, Israel; ^3^Faculty of Social Welfare and Health Sciences, Department of Physical Therapy, University of Haifa, Haifa, Israel; ^4^Department of Diagnostic Imaging, Sheba Medical Center, Tel-Hashomer, Israel; ^5^ARC Innovation Center, Sheba Medical Center, Tel-Hashomer, Israel; ^6^The Division of Data-Driven and Digital Medicine (D3M), Icahn School of Medicine at Mount Sinai, New York, NY, United States

**Keywords:** vestibular pathology, text mining, epidemiologic, diagnostic modalities, trends

## Abstract

**Introduction:**

The field of vestibular science, encompassing the study of the vestibular system and associated disorders, has experienced notable growth and evolving trends over the past five decades. Here, we explore the changing landscape in vestibular science, focusing on epidemiology, peripheral pathologies, diagnosis methods, treatment, and technological advancements.

**Methods:**

Publication data was obtained from the US National Center for Biotechnology Information (NCBI) PubMed database. The analysis included epidemiological, etiological, diagnostic, and treatment-focused studies on peripheral vestibular disorders, with a particular emphasis on changes in topics and trends of publications over time.

**Results:**

Our dataset of 39,238 publications revealed a rising trend in research across all age groups. Etiologically, benign paroxysmal positional vertigo (BPPV) and Meniere’s disease were the most researched conditions, but the prevalence of studies on vestibular migraine showed a marked increase in recent years. Electronystagmography (ENG)/ Videonystagmography (VNG) and Vestibular Evoked Myogenic Potential (VEMP) were the most commonly discussed diagnostic tools, while physiotherapy stood out as the primary treatment modality.

**Conclusion:**

Our study presents a unique opportunity and point of view, exploring the evolving landscape of vestibular science publications over the past five decades. The analysis underscored the dynamic nature of the field, highlighting shifts in focus and emerging publication trends in diagnosis and treatment over time.

## Introduction

Vestibular science, the study of the vestibular system and associated disorders, has garnered increasing attention in the field of medicine over the past five decades. Discernible shifts in topics and trends have emerged, signifying the dynamic nature of vestibulogy as a field of study (1–4).

Epidemiology, diagnostic methods, treatment, and other technological developments have emerged over the years, shifting patient management and outcomes (3–6). Investigating the changing landscape of vestibular disorders across different age groups, from pediatric to geriatric populations, enables the implementation of targeted diagnostic strategies, tailored treatment approaches, and effective preventive measures (2, 3). The etiology of vestibular disorders encompasses a wide spectrum of conditions. Notable conditions such as benign paroxysmal positional vertigo (BPPV), Meniere’s disease, vestibular neuronitis, and vestibular migraine have attracted considerable research interest, leading to the development of innovative diagnostic techniques and therapeutic modalities (5). Understanding the evolving trends in etiological research empowers clinicians and researchers to identify novel diagnostic approaches and explore promising treatment options (4, 5). Accurate diagnosis is a cornerstone in the management of vestibular disorders. Technological advancements have revolutionized diagnostic capabilities, allowing for the utilization of tools such as video head impulse test (VHIT), and computed dynamic posturography (CDP) (6). Analyzing the trends in diagnostic modalities enables a comprehensive evaluation of their efficacy, reliability, and clinical utility, ultimately enhancing the accuracy and precision of vestibular disorder diagnoses (1, 3, 6). Treatment strategies for vestibular disorders have also evolved significantly over time.

To comprehensively explore the trends in vestibulogy over the past five decades, we have employed text-mining techniques. Text mining utilizes computer algorithms to extract valuable information from a vast array of scientific literature, surpassing the capabilities of traditional bibliometric methodologies (1, 3, 6). By employing this approach, we aim to unravel the main trends in published vestibular research, shedding light on emerging research areas and shifts in topics of interest, and to facilitate future investigations.

## Materials and methods

### Dataset

We utilized the US National Center for Biotechnology Information (NCBI) PubMed public application programming interfaces (API) to obtain a comprehensive dataset of relevant publications for our study. The dataset included PubMed unique article IDs (PMID), year of publication, publishing journal, title, keywords, and abstract free-text. The data collection was performed on July 10, 2023, ensuring the inclusion of the most recent available publications up until that date.

### Inclusion criteria

To capture the breadth of research in vestibulogy, we employed a comprehensive search strategy. The search encompassed the titles, abstracts, and keywords of publications. We used the following search terms: (“dizziness,” OR “vertigo,” OR “vestibular disorder,” OR “vestibulopathy,” OR “vestibular hypofunction,” OR “vestibular”) in combination with relevant terms according to each chosen topic. See Table 1 for the full search strategy. The publications considered in our analysis spanned from January 1, 1972 to July 10, 2023. Two otolaryngologists (IT and AW) collaborated to compile the list of terms to categorize the identified publications, ensuring comprehensive coverage across the four broad categories: demographics, etiology, diagnosis, and treatment.

**Table 1:** The terms list used to classify entries for comparison.

Category	Terms
Demographics	Elderly/geriatric/octogenarians/nonagenarians
	Adults
	Pediatric/young/congenital/children
Etiology	Benign paroxysmal positional vertigo/BPPV/otoconia
	Meniere’s disease/endolymphatic hydrops/Meniere’s syndrome
	Vestibular neuronitis/seasonal vertigo/acute unilateral peripheral vestibulopathy
	Superior semicircular canal dehiscence syndrome
	Vestibular migraine/migraine associated vertigo
	Chronic subjective dizziness/ Persistent postural-perceptual dizziness /PPPD/CSD/Phobic postural vertigo/Psychogenic dizziness
	Bilateral vestibular loss/bilateral vestibular hypofunction
	Vestibular schwannoma/acoustic neuroma/acoustic neurinoma/acoustic schwannoma
Diagnosis	Electronystagmography (ENG)/ Videonystagmography (VNG)
	Vestibular evoked myogenic potential (VEMP)
	Rotary chair
	Video head impulse test (VHIT)
	Computed dynamic posturography (CDP)
	Suppression head impulse test (SHIMP)
	Frenzel/video frenzel
Treatment	Physiotherapy/epley/brandt-daroff/bbq/semont/gufoni maneuver/canalith repositioning maneuver/ habituation/adaptation/substitution
	Steroids: Prednisone/steroids/dexamethasone/methylprednisolone
	Medication: Antihistamine/betahistine/meclizine/diuretics/thiazide/uramox/Diamox/gentamycin
	Surgery: Canal plugging/canal resurfacing/middle fossa approach/transmastoid approach/endolymphaic sac decompression/labyrinthectomy/vestibular implant/vestibular nerve section

Main search words: *(Dizziness/vertigo/vestibular disorder/vestibulopathy/vestibular hypofunction) and….*

### Statistical analysis

We conducted all statistical analyses using SPSS (IBM SPSS Statistics for Windows, Version 21.0. Armonk, New York). Descriptive statistics were employed to present categorical variables as counts with percentages unless stated otherwise. Graphical representations were generated using Excel (Microsoft, Version 16.63.1. Redmond, Washington) to visualize the trends identified in the dataset. To determine the publication trend slopes, we fitted linear regression lines to the data, where the year of publication (X) was plotted against the number of publications (Y). We utilized a non-linear regression model to estimate the number of publications in 2023, based on current trends. The model’s accuracy was assessed by calculating the root mean squared error (RMSE), which serves as an indicator of the prediction error. We calculated value of ps for the linear regression lines, with statistical significance set at a two-sided value of *p* < 0.05.

## Results

**Figure 1:**
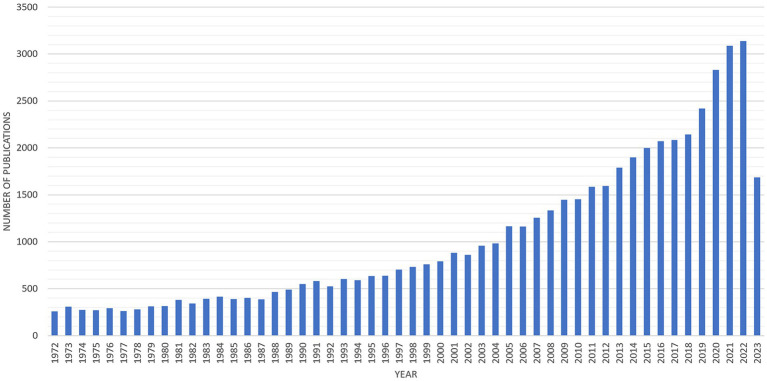
Overall publications trends in vestibular research during the past 5-decades.

A total of 48,916 records were retrieved. After applying the inclusion criteria, 39,238 publications were included in the study cohort. The total publication number by year is presented in Figure 1. According to recent years’ publication trends, the estimated number of publications in 2023 would be 3,254 (±66).

### Topical trends analysis

**Figure 2:**
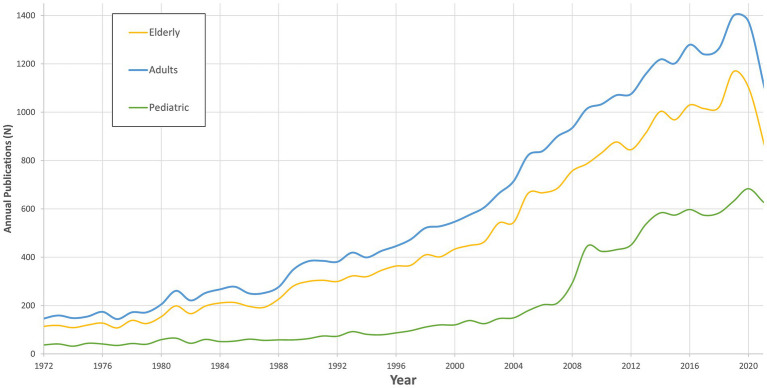
Publications trends in demographic in vestibular research.

**Figure 3:**
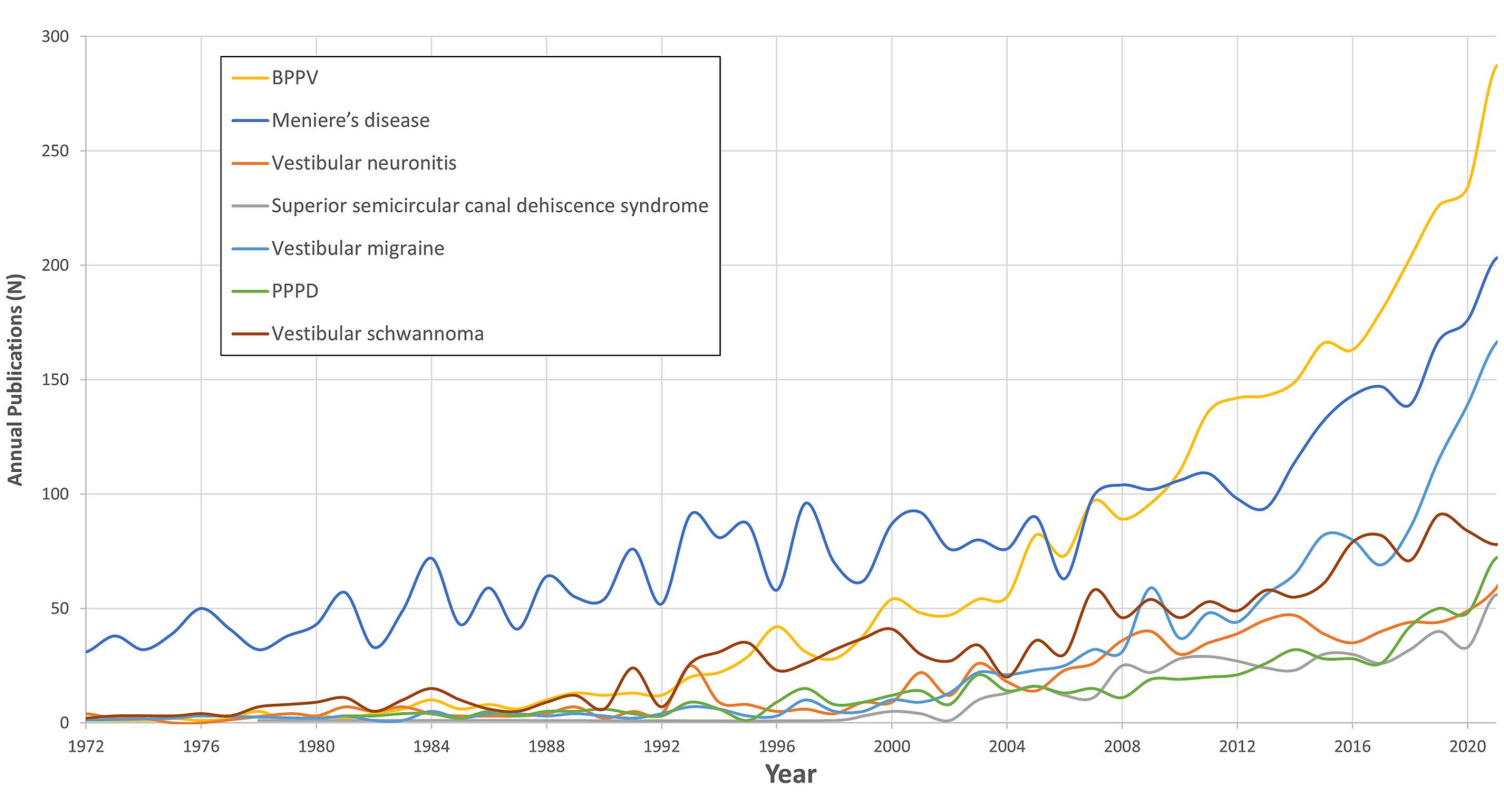
Publications trends in etiological research for vestibular disorders.

**Figure 4:**
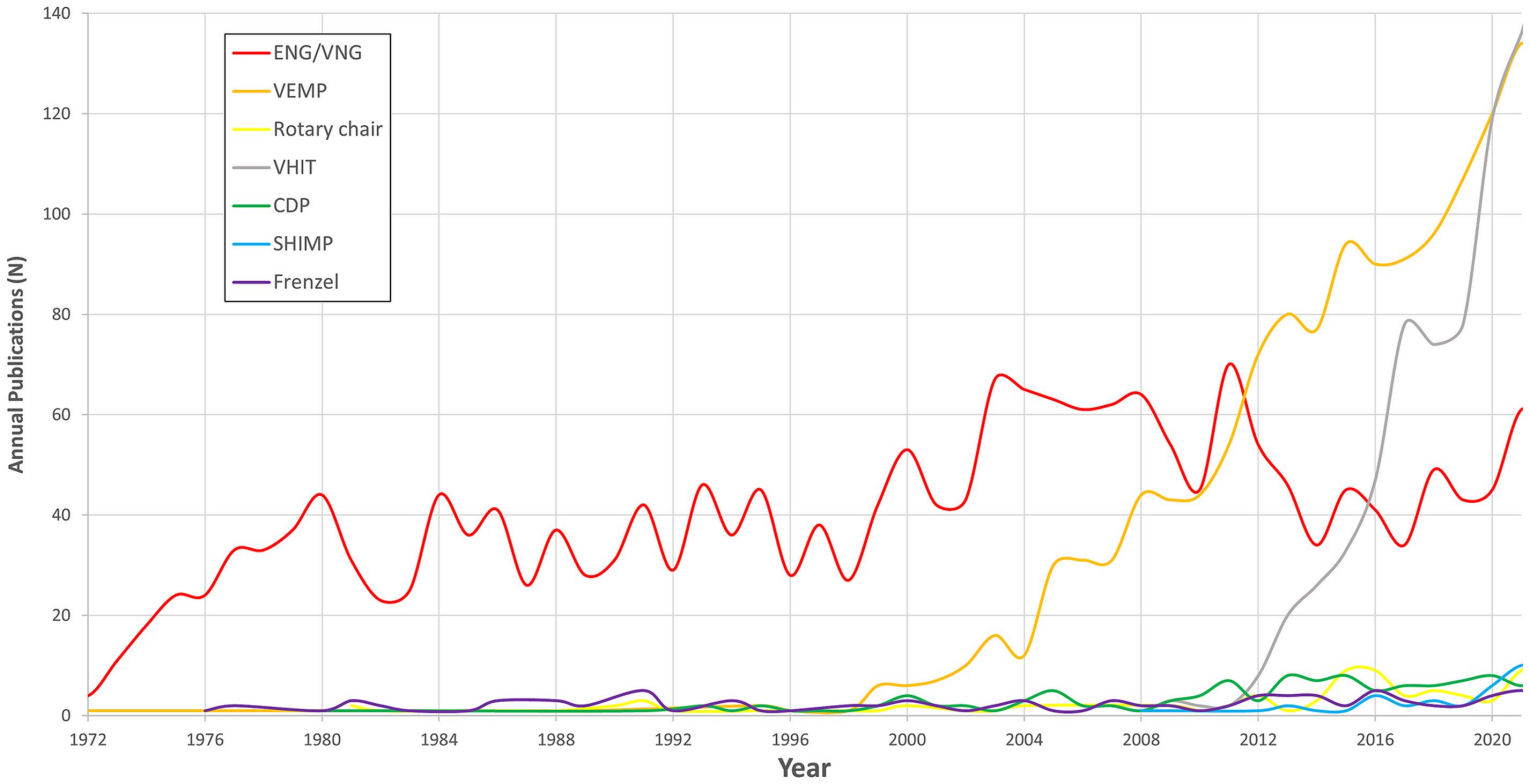
Publications trends in diagnostic method for vestibular disorders.

**Figure 5:**
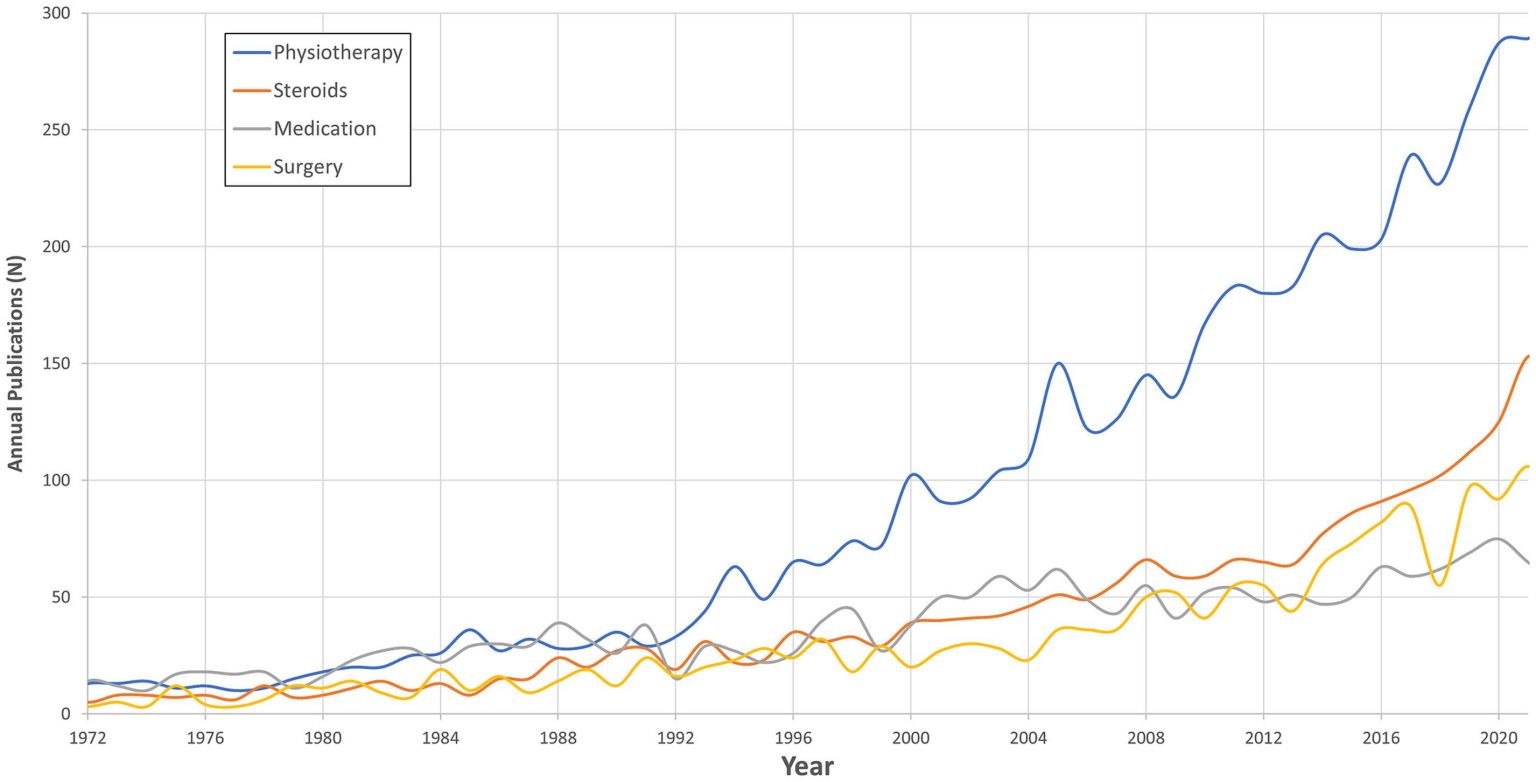
Publications trends in treatment method for vestibular disorders.

The retrieved articles were categorized into the pre-defined research criteria. Specifically, articles were assigned to Demographic (*n* = 32,938), Etiology (*n* = 10,190), Diagnosis (*n* = 3,761), and Treatment (*n* = 9,498). It is notable that several publications fell into two or more categories, reflecting the interdisciplinary nature of the topics. The leading topic was Demographics, representing a significant portion of the overall articles. Publication trends according to the categories are displayed in Figures 2–5.

### Demographics

The overall count of publications focused on the adult population outnumbered the other groups throughout the study period, reaching 31,519 (46.5%) publications. This was followed by the elderly 25,004 (36.86%), while for the pediatric population, the count was 11,315 (16.65%), as shown in Figure 2.

The number of publications on all groups increased over the years, with the highest count of the elderly observed in recent years.

Despite the difference in number of publications, the trends in publication counts for all three age groups showed an upward trajectory. The slope for the elderly was 19.17 (value of *p* <0.001), for adults, it was 23.79 (value of *p* < 0.001), and for the pediatric, it was 12.36 (value of *p* < 0.001). These slopes indicate an increasing research focus on vestibular disorders across all age groups over the past several decades.

### Etiology

The analysis of etiological factors revealed fluctuating patterns in the publication counts related to various vestibular disorders over the study period (Figure 3). BPPV and Meniere’s disease received the most research attention, consistently having higher publication counts compared to other etiologies [3,588 (26.5%) and 4,345 (32.1%)]. In addition to the high publications number, the trend slope of BPPV and Meniere’s disease is the steepest among this group (4.68 and 2.48 value of *p* < 0.001 for both).

These two are followed by Vestibular Neuronitis [1,001 (7.4%)], Superior Semicircular Canal Dehiscence Syndrome (SSCDS) [559 (4.1%)], Vestibular Migraine [1,557 (10.3%)], Chronic Subjective Dizziness/Persistent Postural-Perceptual Dizziness (PPPD) [797 (5.9%)], and Vestibular Schwannoma [1,669 (12.3%)].

Vestibular Migraine and PPPD showed an increase in publication numbers in recent years (1.9 and 0.65, for both *p* < 0.001).

### Diagnostic methods

The highest publication number among the diagnostic techniques was for Electronystagmography (ENG)/Videonystagmography (VNG), with 2,102 (44.01%) publications. This method was followed by Vestibular Evoked Myogenic Potential (VEMP) with 1,473 (30.85%) publications. The Rotary Chair had 88 (1.84%) publications, Video Head Impulse Test (VHIT) had 841 (17.62%) publications, Computed Dynamic Posturography (CDP) had 125 (2.62%) publications, Suppression Head Impulse Test (SHIMP) had 47 (0.98%) publications, and Frenzel goggles had 99 (2.07%) publications.

Examining the entire study period, there were notable shifts in the publications of different diagnostic methods, as illustrated in Figure 4. ENG/VNG publications outnumbered other methods in earlier years, however, with a mild slope of 0.59 (value of *p* < 0.001). VEMP has gained an increasing number of publications, with a rising trend mostly since 2011, reaching a peak of 134 publications in 2021 with a slope of 2.24 (value of *p* < 0.001). VHIT has also seen a substantial increase during these years, becoming the most prevalent method in recent years with a slope of 1.56 (value of *p* < 0.001).

Other diagnostic techniques, such as Rotary Chair and CDP have maintained a relatively steady level of publications over the study period, with varying publication counts. The Rotary Chair had a slope of 0.10 (value of *p* < 0.001), while CDP had a slope of 0.15 (value of p <0.001). SHIMP and Frenzel’s goggles had relatively low publication numbers, with sporadic publication counts across the years. SHIMP had a slope of 0.09 (value of *p* < 0.001), and Frenzel goggles had a slope of 0.06 (value of *p* < 0.001).

### Treatment trends

Overall, physiotherapy (*n* = 5,142, 46.24%) outnumbered all other topics over the years, with the steepest slope of 5.31 (value of *p* < 0.001), as depicted in Figure 5. Medical treatment had a relatively stable publication count over the years (slope: 1.01, value of *p* < 0.001). Steroid treatment, which was considered separately, demonstrated a steeper slope compared to other medical therapies, with a slope of 2.23 (value of *p* < 0.001). Surgical treatment demonstrated a moderate increase, mostly in recent years, with a slope of 1.62 (value of *p* < 0.001).

## Discussion

Our study is a snapshot of the trends in vestibular publications and research in the last 50 years, allowing us to observe some insights about the research community, the disease, and patients’ management throughout these years.

While the preponderance of research has traditionally focused on vestibular disorders in the adult population, our analysis reveals an upward trend in studies across all age cohorts, signaling a broader acknowledgment of the significance of investigating these disorders throughout the lifespan (2, 3). This expanding interest is further illustrated by an increased emphasis on the elderly, a group whose representation in the population is rising. This has been met with a parallel increase in the number of vestibular studies dedicated to this group, similar to other fields (3). Comparatively, publications focusing on pediatric populations account for a smaller proportion of the overall body of work in this field. This discrepancy can be potentially ascribed to the lower rates of prevalence and diagnosis of vestibular disorders in children, combined with the inherent complexities in conducting vestibular assessments in this group (2, 7). Nevertheless, with the recent development of vestibular testing, i.e., simplified, shorter and more physiologic-oriented tests, there is a growing representation of this population in the last decade, including to the younger age groups (8).

Among the tested pathologies, BPPV and Meniere’s disease continue to dominate etiological research within vestibular science, correlating with their higher prevalence (9, 10). Notably, the last decade has seen an accelerated interest in these conditions. This trend reflects not only their prevalence but also their longer historical recognition compared to newer classifications like PPPD or vestibular migraine. Our updated analysis indicates that these pathologies still attract significant research attention, highlighting the enduring importance of their study in the field.

With respect to Meniere’s disease, the incessant quest for enhanced diagnostic modalities and a profound understanding of its pathophysiology to facilitate therapeutic optimization likely accounts for this trend. However, intriguingly, BPPV, a long-standing condition and extensively characterized in the literature, has witnessed a significant resurgence in research interest. This can primarily be attributed to its widespread prevalence in the spectrum of vestibular disorders, particularly among the expanding elderly demographic. In addition, our group and others are constantly searching for a clearer etiology that might help in prevention measures due to the disease burden (11–13). Our study has revealed two additional interesting etiology trends. The first one is vestibular migraine, a topic with limited research that has gained an increasing number of publications in the last two decades. This may be explained by the growing perception of: 1. A better understanding of the impact of migraine and its burden and 2. The association between migraine and vestibular symptoms (14).

We attribute at least part of the recent rising publications rate to the research on the vestibular implant, a possible solution for once-considered incurable medical situations such as bilateral vestibular hyporeflexia due to ototoxicity, bilateral Meniere, CANVAS, or idiopathic (15).

In our view, one topic that has a relatively low publication rate and demands additional attention is PPPD (Persistent Postural-Perceptual Dizziness), a medical condition that is considered the most common chronic vestibular pathology in the 4th–6th decades of life (16). This entity was only formally defined in 2017, and assumed to be related to disruptions in visual processing and postural control mechanisms following vestibular insult (17). The complexity of this condition warrants further research to enhance the understanding and treatment for this pathology.

Diagnostic methods play a critical role in accurate diagnosis and appropriate management of vestibular disorders. Our study reveals shifting trends in the utilization of diagnostic techniques over time. The increasing adoption of Vestibular VEMP and VHIT indicates their growing recognition as valuable diagnostic tools, as they cover the vestibular organs within a physiological frequency range of stimulation (1, 5, 18–20). Conversely, the relatively limited research focus on Suppression Head Impulse Test (SHIMP) and Frenzel goggles highlights the need for further exploration of their diagnostic potential (5).

VNG and ENG demonstrated a mild slop. These are instrumental techniques used to record eye movements as part of vestibular function testing, traditionally including oculomotor and caloric tests. These tests are cumbersome, time-consuming, and relatively expensive tests, which only partially represent the vestibular system in a non-physiologic frequency (21). Nowadays, various systems incorporate VNG and vHIT, which simplify the process and lower the costs. We believe that these trends represent a shift toward more comprehensive, physiologically relevant, fast, and convenient diagnostic methodologies in the field of vestibular disorders, which can drive the field forward.

Our analysis underscores the primacy of physiotherapy as a predominant therapeutic strategy in publications. We believe this suggests its effectiveness and relevance in managing vestibular disorders (2, 22). Publications on medical treatment, including pharmacological interventions, remained stable over time. From our knowledge of research in the field, there is the ambiguity of research outcomes regarding its applicability and need (23, 24). In contrast, we view the increase in publications related to surgical treatment as evidence for the evolving landscape of surgical strategies for vestibular disorders. This trend is particularly relevant in the context of vestibular implants, as previously mentioned (5, 15). Thus, we view the progression of therapeutic modalities in vestibular disorders to be shifting toward the integration of diverse management approaches, including physiotherapy, pharmacology, and evolving surgical interventions.

This study has several limitations. First, our search methodology was restricted to indexing terms and did not extend to the full text of articles. Furthermore, our study analyzes trends without providing individual-based methodological specifics. As such, we do not define an exact age range for our age groups, including the ‘elderly,’ reflecting a broader overview of age-related research trends rather than a precise categorization. This approach may affect age-specific insights but allows for a wider perspective on age group distribution within peripheral vestibular disorder research. Additionally, to avoid over-complexity, the research was limited to peripheral vestibular disorders, thus excluding central vestibular pathologies.

In conclusion, our study presents a unique opportunity to utilize text-mining methodologies to explore the evolving field of vestibular science publications over the past five decades. This analysis provides valuable insights into the current areas of interest within the scientific community, enhancing our understanding of the trends and priorities in vestibular research.

## Data availability statement

The original contributions presented in the study are included in the article/supplementary material, further inquiries can be directed to the corresponding author.

## Author contributions

AW: Conceptualization, Methodology, Writing – original draft. NG: Data curation, Formal analysis, Methodology, Writing – review & editing. YG: Conceptualization, Writing – review & editing. SS: Writing – review & editing. VS: Writing – review & editing. YB: Writing – review & editing. EK: Conceptualization, Writing – review & editing. IT: Conceptualization, Data curation, Methodology, Writing – original draft.
